# The effect of group size and task involvement on temporal binding window in clap perception

**DOI:** 10.3389/fpsyg.2024.1355586

**Published:** 2024-04-24

**Authors:** Takayuki Niizato, Yuta Nishiyama, Keiichi Zempo, Yuka Koike, Koki Arai

**Affiliations:** ^1^Department of Intelligent Interaction Technologies, Institute of Systems and Information Engineering, University of Tsukuba, Ibaraki, Japan; ^2^Department of Information and Management Systems Engineering, Nagaoka University of Technology, Niigata, Japan

**Keywords:** temporal binding window, temporal perception, group size, collective agency, task involvement

## Abstract

We collect various types of information from our environment and organise it to create a coherent representation. Several researchers have suggested that multiple signals within the temporal binding window (TBW) can be integrated into a single coherent experience, such as flashes, beeps, and the McGurk effect. However, there is no evidence that TBW distortion also occurs in group interactions. This study investigates the influence of group size (i.e. the group size effect) and the degree of task involvement in temporal perception using computer-generated clap sound experiments. Participants listened to the randomly generated clap sounds and evaluated whether they were synchronised. We established three conditions based on different levels of task involvement: low (L), middle (M), and high (H) conditions. The varying task involvements reflect how participants interact with the clap sounds, ranging from passive listening in the L condition to actively generating sounds by pressing a key in the M condition, or attempting to synchronise key pressing sounds with the sounds in the H condition. Our experiments show a robust group size effect on TBW, regardless of the different conditions. In other words, as the group size increases, participants perceive the group clap as a single event. Furthermore, we found that the uncertain cause–effect relationship condition (H condition) shows the highest TBW. Interestingly, the TBW in the rigid cause–effect relationship (M condition) is the same as that in the no involvement condition (L condition). Our results suggest that a widened TBW in collective behaviour may facilitate cohesive action, enabling individuals to adapt to the group in relatively uncertain contexts.

## 1 Introduction

We collect various types of information from our environment, and our brain organises it to create a coherent representation (Jagini, 2021). However, this information is often multimodal, with each channel transmitting information at different speeds. To address this challenge, our brain integrates this multisensory information into a single event (Diederich and Colonius, 2004; Vroomen and Keetels, 2010, 2020; Fister et al., 2016; Chen et al., 2018). For instance, a rhythmic synchronisation of the group clap, such as in musical performances, requires the integration of tactile, auditory, and visual information (Vatakis and Spence, 2006). Due to this information binding, the musician perceives the group clap as one unifies event rather than a series of discrete events.

Temporal binding is a factor that combines different events occurring at different times into a single event. Many researchers have demonstrated that several signals within the temporal binding window (TBW) can be integrated into a coherent experience (e.g. the McGurk effect Diederich and Colonius, 2004; Vroomen and Keetels, 2010; Chen et al., 2018). Furthermore, the sense of agency (SOA), which refers to the feeling of being in control of one's actions and their outcomes, influences TBW (Venskus et al., 2021). These findings suggest that temporal binding results from ceaseless predictions about asynchronous stimuli from the environment. These perspectives align with recent Bayesian theory, which states that minimising mismatches between sensory information (prior beliefs) and predicted outcomes leads to updating posterior beliefs. For instance, Shi et al. (2013) showed that outcome predictability can change time perception. Furthermore, Legaspi and Toyoizumi (2019) showed the possibility that time perception can be altered by prediction accuracy.

While only limited research has been conducted on TBW in collective behaviour, the time-shift version of temporal binding, which refers to a shortened subjective time between the cause and effect, provides valuable insights into understanding TBW in a collective context. For instance, Obhi et al. demonstrated that temporal binding occurs through mutual human interactions (Obhi and Hall, 2011a,b). Their results suggest that collective-level agency (i.e., sharing the same agency in the group for the same action Loehr, 2022) also influences temporal perception, particularly when actions can be anticipated, similar to individual agency. Since the time-shift type of temporal binding shares the same mechanism as that proposed by Bayesian theory, it is also reasonable to expect agency-induced TBW.

However, there are two critical differences when applying TBW to group behaviour. First, previous studies have revealed that temporal binding in a group can vary depending on the level of task involvement (Le Bars et al., 2020; Hayashida et al., 2021). For instance, Hayashida et al. (2021) recently found that the degree of temporal binding can change with the presence of a third party. Similarly, Le Bars et al. (2020) found that the method of distributing rewards also alters the degree of temporal binding. These studies indicate that the relationship with other participants is crucial for temporal perception in the group. These results suggest that this tendency can be applied to TBW in group behaviour, where involvement in the task can be a crucial factor in collective behaviour.

Second, in real-world situations, we cannot expect a solid cause-and-effect relationship in group behaviour as we would in previous experimental settings. For instance, in a group clapping scenario, it is challenging to discriminate between the leader and followers as the group size increases (note that this ambiguity is ubiquitous in animal collective behaviour Grégoire et al., 2003; Cavagna et al., 2015; Niizato et al., 2020). This ambiguity requires high task involvement to synchronise timing with other members. Interestingly, studies suggest that these ambiguous cause–effect relationships can enhance coherent collective behaviour, such as mutual anticipation among pedestrians contributing to highly organised group behaviour (Gunji et al., 2012; Murakami et al., 2021; Niizato et al., 2023). However, although some models suggest that cause–effect ambiguity enhances the coherent collective dynamics (Gunji et al., 2012; Murakami et al., 2021; Niizato et al., 2023), empirical collective behaviours are exposed to environmental factors such as group penalties and social rewards (Parrish and Edelstein-Keshet, 1999). Therefore, we must carefully omit these environmental factors to examine the ambiguity of the cause–effect relationship. Our group clap paradigm can address this issue because no environmental cues are present except clapping sounds (i.e. audio perceptual information).

This study examines the impact of group size on TBW under different levels of task involvement. We investigate the group size effect in three conditions: (i) low involvement (pure group size effect), (ii) middle involvement (anticipation effect under a solid cause–effect relationship), and (iii) high involvement (anticipation effect under an uncertain cause–effect relationship). Our experimental design requires participants to evaluate the degree of synchrony in computer-generated clap sounds across each group size and condition. In our experimental setup, we assume that “TBW varies not only with group size but also with the degree of involvement in the task”.

## 2 Materials and methods

### 2.1 Experimental settings

#### 2.1.1 Participants

Twenty adults without any hearing deficit (14 male individuals and 6 female individuals; mean age, 22.7; SD, 2.8) were recruited for Experiment 1, and twenty different individuals who did not participate in Experiment 1 (17 male individuals and 3 female individuals; mean age, 21.5; SD, 1.5) were recruited for Experiment 2. The participants in these two experiments were entirely distinct. The required sample size for each experiment was determined to be over seventeen, based on a power analysis conducted for a 2 × 7 repeated measures ANOVA. This analysis used an alpha of 0.05, a power of 0.80, and an effect size of 0.2, taking into account two factors: ‘task involvement' with two levels, and ‘group size' with seven levels. All participants provided written consent after being informed of the procedures involved. The study was conducted in accordance with the principles of the Declaration of Helsinki and received approval from the Ethics Committee of Tsukuba University (2023R761) and Nagaoka University of Technology (R3-7).

#### 2.1.2 Sound generation

We collected 200 clapping sounds from different individuals. The clap sounds were recorded using a binormal recording apparatus (DR-05; Teac Corp). We generated group clap sounds using these randomly recorded sounds (MATLAB2020b).

We presented clap sounds three times at a frequency of 4 Hz. This frequency was determined based on the rhythm observed when human clapping synchronises (Thomson et al., 2018). Each clap was uniformly distributed within specific intervals, *T*. We set this interval as {0.02, 0.04, 0.06, 0.08, 0.10, 0.12, 0.14, 0.16}. The interval was also randomly selected for a fixed number of groups *M* = {2, 3, 4, 5, 7, 10, 20}. Normalisation was conducted utilising the maximum volume value subsequent to the overlay of clapping noises to mitigate alterations in volume resulting from the superposition of clapping sounds.

For instance, if the group size is *M* = 5 and the interval *T* is 0.04 s, five clap sounds are distributed within this interval uniformly for each group clap. The participant tends to hear the clap sounds as one because the individual claps within small intervals are dense. In contrast, when the interval *T* is large, such as 0.16 s, the participant hears the discrete claps rather than the group clap. We have listed the sample sounds used for Appendix S3.

### 2.2 Procedure

Participants wearing noise-cancelling headphones and an eye mask to block their sight sat in front of a keyboard connected to the PC. They heard a sound stimulation generated using MATLAB software. Noise-cancelling headphones mitigated the noise. The stimulus presentation and data acquisition were performed using Psychtoolbox-3 (Kleiner et al., 2007) in MATLAB2020b.

#### 2.2.1 Experiment 1

**Figure 1:**
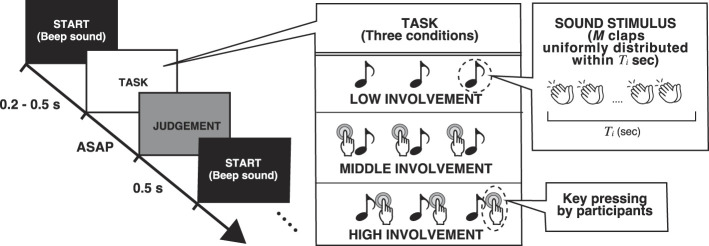
Schematic of the experimental procedure. **Left**: Participants judging the synchrony of the group clapping. In the low (L) condition, the subject passively listens to the auto-generated sounds at 4 Hz. In the middle (M) condition, the subject generates clap sounds by pressing a key at approximately 4 Hz. In the high (H) condition, the subject aims to align with the auto-generated sound by pressing the key. **Right**: The detail of the note symbol from the left scheme. The clap sounds were uniformly distributed within *T*_*i*_ s, where *T*_*i*_ was randomly selected from {0.02, 0.04, 0.06, 0.08, 0.10, 0.12, 0.14, 0.16}. *i* represents the trial number.

To observe the effect of the group size for group clapping, we conducted an experiment using a full range of *M*, where participants judged the synchronicity of group clapping sounds. This experiment included two conditions. Under the low involvement condition, three group clap sounds (intervals of 4 Hz) were automatically generated from 0.2 s to 0.5 s after a mask sound (beep lasting 0.2 s). In the middle condition, participants generated the sounds by pressing the “ENTER" key three times at 4 Hz. Therefore, the middle condition (i.e. listening to and generating the clap sound) required higher involvement from the participant compared to the low condition (i.e. listening to the clap sound). If the agency widens the TBW, the subject of the middle condition is expected to show a wide TBW compared with the low condition.Figure 1

Participants performed seven sessions with different group sizes in either low or middle conditions (7 × 2 sessions in total). Each session consisted of 160 trials (20 trials for each *T*) after 20 training trials. The participants were asked to answer “Is the clap sound almost in sync?” by pressing a key every trial. Pressing the key “RIGHT SHIFT" represented a positive answer, YES, and “LEFT SHIFT" represented a negative answer, NO. After a session, they rested for 5 min and proceeded to another session. Experiments conducted on 5 different days were combined for results because, at most, three sessions were performed in a day to avoid fatigue. The order of the group sizes was random for each participant, and the order of the active and passive conditions was counterbalanced.

#### 2.2.2 Experiment 2

This experiment was conducted using the same procedure as that in Experiment 1 but without the low condition and with varying group sizes. Instead of the low condition, we used a high involvement condition, where participants were asked to press the ENTER key every time they heard the group clap sounds generated automatically at 4 Hz. Note that their key press generated nothing and just followed the sounds immediately after they heard them. In other words, the participants' intention to follow the sounds was active, but their action to be performed was passive.

We predicted that the high condition (i.e., listening, generating, and attempting to align to the clap sound) would create a situation of higher involvement than both the middle and low conditions (i.e. listening and generating the clap sound). In general, people involved in group clapping are embedded in an uncertain causal relationship. Each person is unable to discern whether they are leading or following in the group clapping. Thus, the participant under the group interaction must adjust their clap timing within this uncertain context. This process could generate a high degree of TBW in the high condition. Unlike in Experiment 1, we set the group size *M* to be between 2 and 5 in Experiment 2 because a wide range of *M* is not necessary to investigate differences in perception between conditions.

### 2.3 Data analysis

After the experiment, we estimated how the participants perceived group clapping sounds at an individual level. Following the traditional method, we fitted the obtained data to a sigmoidal function.


(1) $$\begin{array}{l} y\left(\tau \right)=\frac{1}{1+\exp^{-\frac{\left(\tau -a\right)}{b}}} \end{array}$$

where τ is the independent variable (i.e. clap time intervals), and *y* is the dependent variable (i.e. the probability of sound simultaneity judgement *n*/20 for each τ). When τ equals zero, the group clapping is completely synchronised; *y*(τ = 0) is expected to be one. By contrast, *y*(τ = ∞) is zero as no simultaneity judgement is expected.

**Figure 2:**
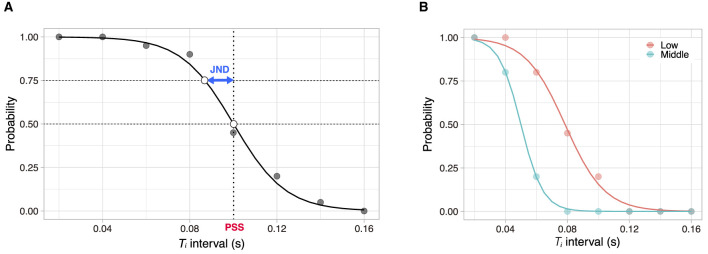
Sample of an experimental data and its sigmoidal fitting. **(A)** The dots represent empirical samples from our experiment, and the solid line represents the best-fit sigmoidal function. In this case, the parameters are *a* = 0.101 and *b* = 83.9. PSS can be defined as the point at *y*^−1^ (0.5). JND can be defined as the difference between *y*^−1^ (0.5) and *y*^−1^ (0.75). In this example, PSS is 0.101 (s), and JND is 0.013. **(B)** An example of sigmoidal fitting for different task involvements (Experiment 1).

Moreover, the parameter *a* is known as “the point of subjective simultaneity” (PSS) in psychophysics, and the parameter *b* is the slope of the sigmoid function, which is related to another psychophysical value, as mentioned below. We used MATLAB approximation for the fitting. Data with a root mean error (*R*^2^) of less than a particular value (*R*^2^ <0.5) were excluded from the analysis because a sigmoidal model does not adequately fit with such data (Serino et al., 2018). Figure 2 shows an example of the sigmoidal fitting to the empirical samples.

The PSS (i.e. *a*) is the point at which *y*(τ) equals 0.5. At this point, the participant could not judge whether the stimulation was grouped into two extremes. The PSS, in this sense, is the limit of the participant's discrimination ability. We considered the PSS as an index of the TBW limit of different stimuli (individual claps) as one stimulus (group clap).

In addition, we estimated the critical value, “just noticeable difference” (JND), which is derived from the ambiguity of the participant's judgement. The JND depends on the slope parameter *b*. In this study, we defined JND as *y*^−1^(0.75)−*y*^−1^(0.5). A small value indicates a distinct judgement, whereas a large value indicates a vague judgement.

Generally, PSS is defined as the limit of distinguishability of a given stimuli (Paraskevoudi and Vatakis, 2019); however, some researchers relate this value to TBW, which is equivalent to the PSS ± JND (Vatakis et al., 2008; Keetels and Vroomen, 2012; Benedetto et al., 2018). PSS represents the mean TBW, and JND represents its standard deviation from PSS. In this context, we apply PSS and JND to measure the temporal perception (i.e. TBW). Furthermore, PSS can change under different experimental conditions, such as cognitive loads (De Niear et al., 2016; Chiu et al., 2019) and combinations of modalities (Vatakis et al., 2008; Navarra et al., 2013; Benedetto et al., 2018). Therefore, this study hypothesises that PSS also varies with the experimental conditions including the group size and task involvement in collective behaviour.

## 3 Results

### 3.1 Experiment 1

Data of three participants were excluded from the analysis following the criteria *R*^2^ <0.5.

We hypothesised that PSS varies with group size and task involvements. A 2 × 7 repeated measures ANOVA (RM ANOVA) design was employed for the PSS, examining two levels of task involvement (L and M) across seven group sizes. The RM ANOVA was implemented subsequent to the execution of Mendoza's multisample sphericity test (*p* = 0.0130) and the epsilon correlation assessment. Further details can be found in Supplementary Table S1. The main effect of group size was significant (*F*(3.63, 58.12) = 16.50, *p* < 10^−5^, ω^2^ = 0.0783). In contrast, the main effect of the degree of involvement was not significant (*F*(1, 16) = 3 × 10^−4^, *p* = 0.99, ω^2^ = −0.0049). Additionally, the interaction was not significant (*F*(6, 96) = 0.74, *p* = 0.62, ω^2^ = −0.0007). Note the negative values of omega squared (ω^2^) are mathematically possible but uncommon, often indicating a small sample size or overestimation of the model. These values suggest that the effects are negligible or absent. For the subsequent analyses, the interpretation of ω^2^ remains consistent with this understanding.

**Figure 3:**
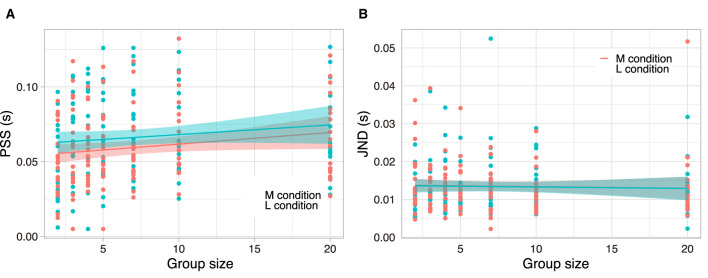
PSS (left) and JND (right) for the L (blue) and M (red) conditions. **(A, B)** The dots indicate all PSS and JND data for 17 participants. The solid line represents the linear fitting, and the shaded colour is the confidence interval 95%.

The trends are illustrated in Figure 3A. The graph also shows the proportional relationship between group size and PSSs for both degree of involvements (for *post-hoc* comparisons with t-test, see Supplementary Table S2). This result suggests that the participants tend to weaken the criterion of the judgement of clapping in sync with increasing group size. The results of independent conditions indicate that this tendency in simultaneous judgements is robust (see Supplementary material).

We hypothesised that JND varies with group size and task involvements. A 2 × 7 RM ANOVA design was employed for the JND, examining two levels of task involvement (L and M) across seven group sizes (Figure 3B). The RM ANOVA was implemented subsequent to the execution of Mendoza's multisample sphericity test (*p* = 0.5108) and the epsilon correlation assessment. Further details can be found in Supplementary Table S1. The main effect of group size was not significant (*F*(6, 96) = 3.12, *p* < 0.1, ω^2^ = 0.0152), nor was the main effect of the task (*F*(1, 16) = 1.89, *p* < 0.1, ω^2^ = 0.0109), and the interaction was also not significant (*F*(6, 96) = 1.48, *p* = 0.19, ω^2^ = 0.0087). Therefore, despite the group effect on PSS, JND never shows the group effect. This result suggests that a widened PSS has little effect on judgement ambiguity (i.e., JND).

### 3.2 Experiment 2

In Experiment 1, we observed variability in the PSS relative to the group size; however, we did not discern any significant differences in PSS attributable to task involvement. Consequently, we further explored the potential for detecting the effect on the task level by augmenting the complexity, that is, the ambiguous cause–effect relationship between the participant's action (key press) and the generated clapping sound.

Data of a participant were excluded from the analysis following the criteria *R*^2^ <0.5.

Unlike in Experiment 1, we set the group size *M* to be between 2 and 5 in Experiment 2 because a wide range of *M* is not necessary to investigate differences in perception between conditions. We hypothesised that PSS varies with group size and task involvement. A 2 × 4 RM ANOVA design was employed for the PSS, examining two levels of task involvement (M and H) across four group sizes. The RM ANOVA was implemented subsequent to the execution of Mendoza's multisample sphericity test (*p* = 0.0003) and the epsilon correlation assessment. Further details can be found in Supplementary Table S1. The main effect of the group size was significant (*F*(1.96, 37.18) = 47.6, *p* < 10^−5^, ω^2^ = 0.277), as was the main effect of the degree of involvement (*F*(1, 19) = 26.4, *p* = 10^−4^, ω^2^ = 0.1601). However, the interaction was not significant (*F*(3, 57) = 1.43, *p* = 0.242, ω^2^ = 0.0019).

**Figure 4:**
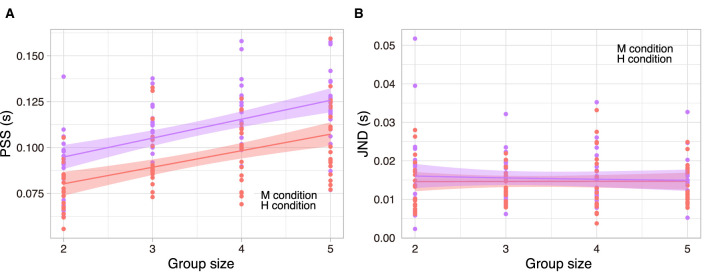
PSS (left) and JND (right) for the M (red) and the H (purple) condition. **(A, B)** The dots indicate all PSS and JND data for 19 participants. The solid line represents the linear fitting, and the shaded colour is the confidence interval 95%.

Figure 4A shows the proportional relationship between group size and PSSs (for *post-hoc* comparisons with t-test, see Supplementary Table S3). However, compared to Experiment 1, the two lines did not overlap. As noted earlier, the PSS in the H condition was significantly higher than that in the M condition, indicating that the participants perceived the group clap as more synchronised in the H condition than in the M condition.

We hypothesised that JND varies with group size and task involvement. A 2 × 4 RM ANOVA design was employed for the JND, examining two levels of task involvement (M and H) across four group sizes (Figure 4B). The RM ANOVA was implemented subsequent to the execution of Mendoza's multisample sphericity test (*p* = 0.059). The main effect of group size was not significant (*F*(3, 57) = 0.35, *p* = 0.79, ω^2^ = −0.0099), nor was the main effect of task (*F*(1, 19) = 0.90, *p* = 0.36, ω^2^ = −0.0003), and the interaction was also not significant (*F*(3, 57) = 0.28, *p* = 0.84, ω^2^ = −0.0077).

Intriguingly, the PSS obtained under the high (H) condition was observed to be significantly larger than those obtained under the medium (M) condition; however, this trend was not mirrored in the JND. Participants increased their perceived synchrony (i.e. PSS) across different group sizes without a corresponding increase in the ambiguity of their judgements (i.e., JND). These results imply that a widened PSS does not inherently diminish judgment precision.

## 4 Discussion

Our group clapping experiment reveals two key findings. First, TBW (i.e., PSS±JND) is proportional to the group size, irrespective of task involvement. We refer to this effect as “the group size effect,” which is solely dependent on the size of the group. The main cause of this effect is the increased density between claps within a given period. Furthermore, we also suggest the possibility of a logarithmic relationship, rather than a linear one, between TBW and group size (Supplementary Figure S2). While we do not assert that the logarithmic relationship is definitively valid, this logarithmic relationship has advantages in understanding a particular collective behaviour as we will discuss later. Second, the widened TBW was observed only in the H involvement condition. The most straightforward explanation for this observation is the increased cognitive load (De Niear et al., 2016); however, two factors prevent the cognitive load interpretation. First, no significant difference was observed despite the differences in load between the M and L conditions. Second, our findings suggest that the JND (i.e., indicative of judgement ambiguity) remains unaffected by both group size and task involvement. This result may be important because a previous study has shown that JND increases when the cognitive load increases (Chiu et al., 2019). Therefore, we cannot explain the reason for the increasing PSS via the divided attention considering the task complexity. As a result, we must consider that additional factors other than cognitive load affect this system. These two issues are discussed as follows.

The group size effect suggests that individuals within a group are more likely to perceive a sense of unity in the same action. However, we need to consider where this effect contributes to real collective behaviour. One possible indication is the phenomenon of joint rushing in the group's rhythmic behaviour. It is well-known that the rhythmic tempo (or frequency) tends to increase in a large group size. Even the trained musical performer can drag their tempo without an external cue (Wolf et al., 2019). Recent studies have revealed the group size dependency for the group rhythmic interaction (Thomson et al., 2018; Wolf et al., 2019; Wolf and Knoblich, 2022). Thomson et al. (2018) showed that the simple frequency tuning model, which aligns an individual's frequency with the average frequency of the group, cannot replicate the group size-dependent joint rushing phenomena.

However, we found that employing the logarithmic function to describe the relationship between the group size and TBW allows us to replicate the group size-dependent joint rushing without any additional assumption (Supplementary Figure S3). In other words, the extended TBW with respect to the group size can facilitate group synchrony. If our results can be applied to the general collective behaviour, it may be necessary to reevaluate certain modelling assumptions. For instance, our result will cast doubt on the uniform updating rule, which assumes the existence of a global clock and all individuals acting simultaneously (Gunji et al., 2012) because the PSS can differ under various conditions.

Next, we consider the other possibility for the widened TBW observed exclusively in the H condition. As previously discussed, if the cognitive load was the primary factor driving TBW distortion, we would expect to see differences between the L and M conditions. Furthermore, applying Bayesian inference, the highest agency, such as the participant in the M condition who can generate a solid cause–effect relationship, would also contribute to widening the TBW (Jazayeri and Shadlen, 2010; Shi et al., 2013; Legaspi and Toyoizumi, 2019). However, our results show the opposite. The highest TBW in the H condition suggests that the ambiguous cause–effect relationship (unlike that in the M condition) contributes the most to TBW changes.

We note that the H condition reflects the actual group interaction. The agent cannot discriminate the cause–effect relationship in real-world situations as no linear causal relationship exists in the group (Gunji et al., 2012; Murakami et al., 2017). At times, agents may take action before others, while in other instances, they may follow the actions of their peers. The agent cannot consistently be the true cause of the group's collective action. Our result suggests that this uncertainty enhances the coherent group action by extending the TBW (Supplementary Figures S2, S3).

The widened TBW in this uncertain cause–effect relationship can be interpreted in the context of adaptive behaviour as a causal relationship is not predetermined but created. For example, Cavazzana et al. (2014) suggested a bidirectional relationship of causality in childhood. Blaky et al reported that the temporal binding sore tends to be high between the ages of 4 and 6 (Blakey et al., 2019; Aytemur and Levita, 2021). Furthermore, as discussed in the Introduction, the temporal biding rate also changes with the social context (Le Bars et al., 2020; Hayashida et al., 2021). Therefore, the causal relationship may be a product of human adaptability in social relationships. Consistent with this notion, the relatively widened TBW in the H condition suggests that participants expand their temporal window to adapt to the group behaviour in a weakly predictable (periodic) context. In other words, the uncertain relationship promotes group synchrony as a form of social adaptability.

In our study on clap sound perception, we encountered methodological limitations, notably in the instructions provided, sound distribution, and evaluation methods. Regarding sample size, the modest number of participants warrants careful interpretation of our results, highlighting the need for a larger sample to ensure statistical robustness in future research. Furthermore, we allowed participants to adjust the volume to their own comfort level, but we did not record specific decibel values. This approach enabled us to focus our analysis on how temporal binding and group size influence perception. This approach limited the impact of individual volume preferences on the study's outcomes but also omitted the examination of absolute volume levels. Future studies should consider precise volume measurements to explore their potential effects on clap sound perception.

## Data availability statement

The original contributions presented in the study are publicly available. This data can be found here: https://github.com/t-niizato/The-group-size-effect-of-temporal-binding-window-on-clap-perception.

## Ethics statement

The studies involving humans were approved by the Ethics Committee of Tsukuba University (2023R761) and Nagaoka University of 216 Technology (R3-7). The studies were conducted in accordance with the local legislation and institutional requirements. Written informed consent for participation in this study was provided by the participants' legal guardians/next of kin.

## Author contributions

TN: Writing—review & editing, Writing—original draft, Visualization, Supervision, Software, Resources, Project administration, Methodology, Investigation, Funding acquisition, Formal analysis, Data curation, Conceptualization. YN: Writing—review & editing, Writing—original draft, Visualization, Validation, Supervision, Resources, Project administration, Methodology, Formal analysis, Data curation. KZ: Writing—review & editing, Validation, Project administration, Methodology. YK: Writing—review & editing, Validation, Methodology, Formal analysis, Data curation. KA: Writing—review & editing, Validation, Methodology, Formal analysis, Data curation.
