# Supplementary material for: The effect of group size and task involvement on temporal binding window in clap perception
Source: Front Psychol. 2024 Apr 24;15:1355586. doi: 10.3389/fpsyg.2024.1355586 (PMC11076830; doi:10.3389/fpsyg.2024.1355586)
Supplement: Supplementary file 2 [file Data_Sheet_2.pdf]

Supporting Information

Participants

Twenty adults without any hearing deficit (18 males and 2 females; mean age, 22.8; SD, 2.6) were recruited for this experiment. All participants provided written consent after receiving an explanation of the procedures involved. The study was conducted in accordance with the principles of the Declaration of Helsinki and approved by the Ethics Committee of Tsukuba University (2023R761) and Nagaoka University of Technology (R3-7).

Sound Generation

We collected 200 clapping sounds from different individuals. The clapping sounds were recorded using a binormal recording apparatus (DR-05; Teac Corp.). We generated group clapping sounds using these randomly recorded sounds (MATLAB2020b).

We presented the clapping sounds three times at a frequency of 4 Hz. This frequency was determined based on the rhythm observed when human clapping synchronizes [1]. Each clap was uniformly distributed within specific intervals, T. We set this interval as $\{0.02, 0.04, 0.06, 0.08, 0.10, 0.12, 0.14, 0.16\}$. The interval was also randomly selected for a fixed number of groups $N = \{2, 3, 4, 5, 7, 10, 20\}$. Normalisation was conducted utilising the maximum volume value subsequent to the overlay of clapping noises to mitigate alterations in volume resulting from the superposition of clapping sounds.

Procedure

This experiment was carried out using the same procedure as that in Experiment 1, except for an instruction for participants to answer, "Is the clap sound in sync?" We used this instruction, omitting the word "almost" from

the question used in Experiments 1 and 2, to allow the participants make stricter judgements.

Experiment 1': Low and Middle Conditions

Participants answered the question "Is the clap sound in sync?" for each group (160 trials after 20 training trials). This process was conducted in a single session. If the participants provided a "YES", they pressed the "RIGHT SHIFT" key; otherwise, they pressed the "LEFT SHIFT" key. Under the Low (L) condition, sound was generated automatically after masking (beep sound). In the Middle (M) condition, the subject pressed the "ENTER" key three times at 4 Hz. The button press generated clap sounds.

In contrast to the L condition, the participants could produce group sounds. After the session, each subject rested for 5 min and underwent another session (different group sizes). To avoid fatigue, each participant underwent a maximum of three sessions daily. The order of the group sizes was random for each participant. The order of the M and L conditions was counterbalanced.

Results

Data of two participants were excluded from the analysis following the criterion $R^2 < 0.5$.

We hypothesized that PSS varies with group size and task involvements. A 2×7 RM ANOVA design was employed for the PSS, examining two levels of task involvement (L and M) across seven group sizes. The RM ANOVA was implemented subsequent to the execution of Mendoza's multisample sphericity test ($p = 0.0106$). The main effect of the group size was significant ($F(3.54, 60.12) = 8.78, p < 10^{-5}, \omega^2 = 0.0348$), and the main effect of conditions was also significant ($F(1, 17) = 5.06, p < 0.05, \omega^2 = 0.0094$). However, the interaction was not significant ($F(6, 102) = 1.10, p = 0.37, \omega^2 = 0.0003$).

Figure S1 shows that the proportional relationship of PSS is maintained in this condition despite the different instructions reducing the overall magnitude of PSS compared to those in Experiments 1 and 2. This suggests that this tendency is robust for the perception of group clapping.

We hypothesized that JND varies with group size and task involvements. A 2×7 RM ANOVA design was employed for the JND, examining two

levels of task involvement (L and M) across seven group sizes. The RM ANOVA was implemented subsequent to the execution of Mendoza's multisample sphericity test ($p = 0.0012$) and the epsilon correlation assessment. The main effect of group size was not significant ($F(3.41, 57.9) = 0.204, p = 0.91, \omega^2 = -0.0113$), and the main effect of task was not significant ($F(1, 17) = 0.03, p = 0.87, \omega^2 = -0.0021$). However, the interaction was significant ($F(6, 102) = 3.35, p = 0.0047, \omega^2 = 0.0171$). A post hoc test showed significant differences on $N = 2$ ($F(1, 17) = 5.72, p = 0.0287, \omega^2 = 0.072$) and $N = 7$ ($F(1, 17) = 5.72, p = 0.032, \omega^2 = 0.0453$).

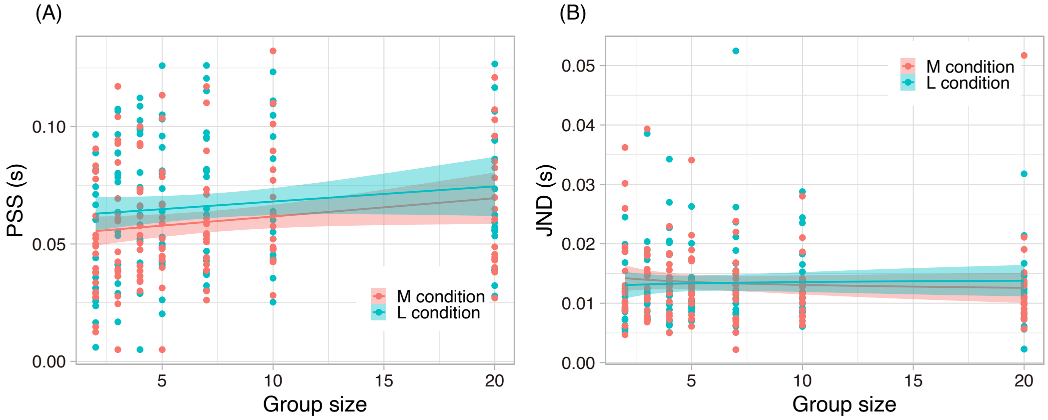

Figure S1: **PPS (left) and JND (right) for the L (blue) and M (red) conditions.** (A, B) The dots indicate all PSS and JND results for 18 participants. The solid line represents the linear fitting, and the shading colour shows the confidence interval 95%.

Weber Fraction

Although we have confirmed the proportional relationship between the PSS and group size, we found that a logarithmic relationship better fits the empirical data and is more suitable for the Akaike (or Bayesian) information criterion. This logarithmic relationship suggests that our findings may be related to Weber-Fechiner's law in the TBW.

The Weber fraction (Wf) is the ratio of a just-noticeable difference (JND)

to intensity (I), and the Weber constant is k. However, recent research has indicated that (Wf) is not necessarily constant[2, 3, 4].

We apply Haigh's definition Wf [4] as follows:

$$Wf = \frac{JND}{PSS}$$

This equation indicates that if Wf is constant, the stimulus intensity (i.e. PSS) increases with perceptual discrimination (i.e. JND). Therefore, the smaller the Wf, the better the stimulus-change noticeability for the subject, and the higher the Wf, the poorer the stimulus-change noticeability for the subject.

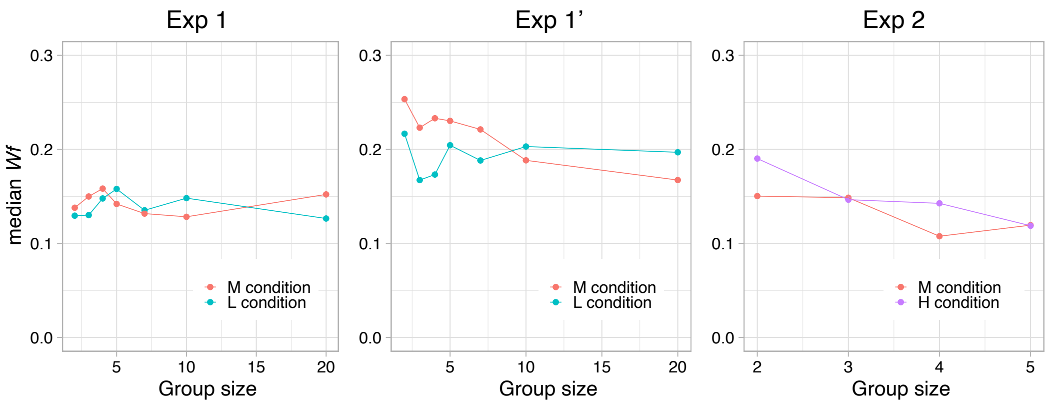

Figure S2: **Weber fraction (*Wf*) for each experiment.** Blue indicates the L condition, red indicates the M condition, and purple indicates the H condition.

Figure S2 shows the Wf for the three experiments. Each point represents the median of Wf (to reduce the effect of outliers) for group size.

The Wf in Experiment 1 shows the main effect of the group size (RM ANOVA design: $F(6, 96) = 2.48, p < 0.05$). The primary effect of the condition was not significant (RM ANOVA design: $F(1, 16) = 2.52, p = 0.13$), and the interaction was not significant (RM ANOVA design: $F(6, 96) = 2.03, p = 0.07$). In contrast, the Wf in Experiment 1' showed no major influence of the group size (RM ANOVA design: $F(1, 102) = 1.26, p = 0.28$), the condition (RM ANOVA design: $F(1, 17) = 0.35, p = 0.56$), or the interaction (RM ANOVA design: $F(6, 102) = 0.19, p = 0.38$). The Wf in

Experiment 2 showed the primary effect of the group size (RM ANOVA design: $F(3, 57) = 3.67, p < 0.05$) and the condition (RM ANOVA design: $F(1, 19) = 2.52, p < 0.05$). However, the interaction was not significant (RM ANOVA design: $F(3, 57) = 0.32, p = 0.81$).

These findings have two essential implications. First, Weber's law does not hold in time perception. The condition difference in Weber's law is confirmed only in H mixed conditions. In this context, an uncertain environment may affect Weber's law. Second, because a large Wf leads to low temporal resolution, a large Wf in an uncertain context may enhance the unified group clap.

Emergence of Joint Rushing

In this section, based on the logarithmic relationship governing the perception of group clapping discussed previously, we revisit the mathematical model that failed to explain the joint rushing phenomenon empirically found in the previous study [1]. Consider M agents indexed by i, each with phase $x_i \in [0, 1]$ and a frequency of $\frac{dx_i}{dt} = \omega_i$. If x_i reaches 1 (indicating the agent i's clap), it is reset to 0, and the frequency is then discretely updated via

$$w_i(t + \Delta t) = \xi_i + \frac{1}{T_{\text{avg}} + t_{\text{avg,last}} + T_{\text{avg}} - t} \tag{1}$$

ξ_i is the Gaussian error with the mean μ and standard deviation σ, determining the individual tendency of the frequency. $T_{\text{avg}} = \frac{1}{N} \sum_{j=1}^{N} \frac{1}{w_j(t)}$ is the average group sound period, $t_{\text{avg,last}}$ is the average last clap time for the M individuals, and t is a clap time from the start of the simulation. Thus, $t_{\text{avg,last}} + T_{\text{avg}} - t$ shows a mismatch between the individual clap time and the expected group clap time. Consequently, the frequency $w_i(t + \Delta t)$ is updated to minimise this mismatch. If the mismatch is negative (indicating that the clap timing is delayed), the frequency increases. In contrast, if it is positive (indicating the clap timing is forwarded), the frequency decreases.

Thomson et al. [1] concluded that this model failed to explain the joint rushing phenomenon as the frequency slope did not increase with group size. Their model has a fixed Δt at 0.0025 s, assuming a minimum time required to adjust the next clapping time. However, it is reasonable to vary Δt if we regard it as the time taken to recognise the synchronised group clapping (PSS), as we found in our experiments. Therefore, we conducted 10000 tests

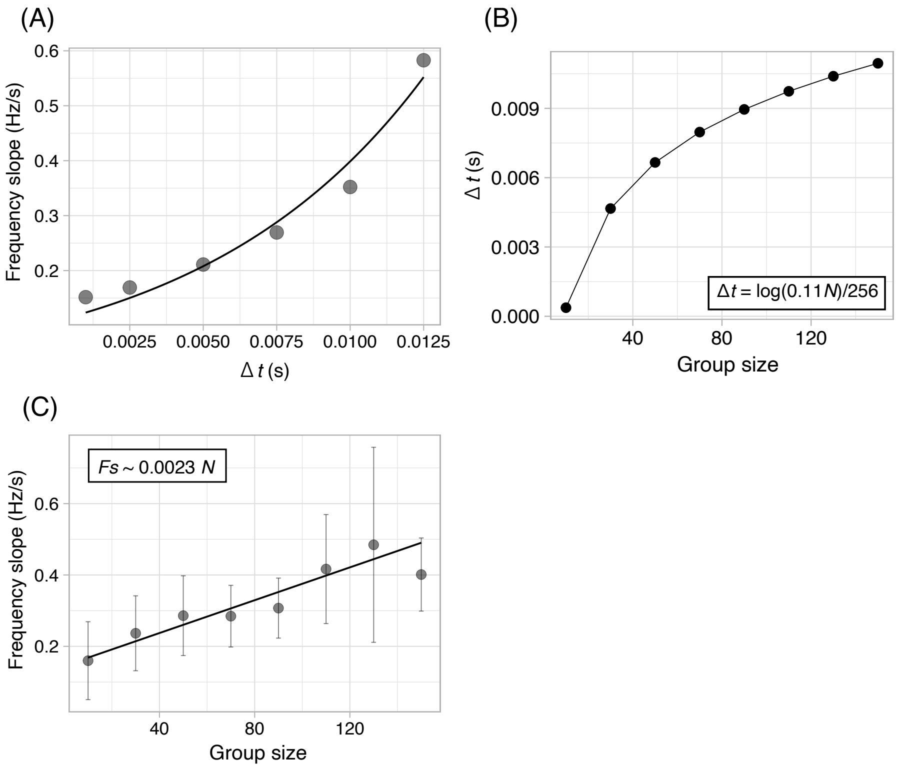

Figure S3: **Joint rushing model with the Weber–Fechner law**. Simulations were performed with $\mu = 0.05$ and $\sigma = 0.1$ following a previous model [1]. (A) Frequency slope variant to Δt. The black line shows the exponential approximation. (B) Theoretically derived Weber–Fechner relationship between Δt and the group size M. (C) Frequency slope varies with the group size when applying the Weber–Fechner relation.

for each dataset and determined the frequency slope by varying Δt. The parameter settings for other details were the same as those used in the study by Thomson et al. [1]. As a result, we found that the slope of frequency increase to Δt was fitted to an exponential function $fs = e^{\Delta t}$ (Figure S3A). Considering the experimental findings in the previous study [1], the frequency rate is variant with the group size with $fs = cN$, where c represents a constant. Thus, the relationship between Δt and the group size can be derived theoretically as $\Delta t = \log(cN)$ (FigureS3B). This finding aligns with

our observation of the logarithmic function describing the group clapping perception, although the magnitude of the values may differ depending on the experimental settings. Finally, this relationship successfully reproduces Thomson's experimental results, demonstrating that varying group size has an effect on the frequency slope (FigureS3C).

References

[1] Michael Thomson, Kennedy Murphy, and Ryan Lukeman. Groups clapping in unison undergo size-dependent error-induced frequency increase. *Scientific reports*, 8(1):808, 2018.

[2] Sergio Cesare Masin, Verena Zudini, and Mauro Antonelli. Early alternative derivations of fechner's law. *Journal of the History of the Behavioral Sciences*, 2009.

[3] Lewis A. Bizo, Josey Y.M. Chu, Federico Sanabria, and Peter R. Killeen. The failure of Weber's law in time perception and production. *Behavioural Processes*, 2006.

[4] Andrew Haigh, Deborah Apthorp, and Lewis A. Bizo. The role of weber's law in human time perception. *Attention, Perception, ¥ Psychophysics*, 83:435–447, 2020.

Supporting Tables

To test differences between three conditions, we applied RM ANOVA and post-hoc comparisons with t-test corrected using extended Bonferroni procedure [1] to obtained data below. The significance level was set at $p < .05$. Data were analyzed with statistics software (R, The R Foundation for Statistical Computing, Vienna, Austria).

[1] J.P. Shaffer, "Modified Sequentially Rejective Multiple Test Procedures", Journal of the American Statistical Association, Vol. 81, No. 395, pp. 826-831, 1986.

Table S1. Mendoza's Multisample Sphericity Test and Epsilons for each Experiment.

Mendoza's Multisample Sphericity Test and Epsilons of group size for df correction

Effect	Lambda	approx.Chi	df	p	LB	GG	HF	CM
Group size (Exp1: PSS)	0	30.5361	20	**0.0674**	0.1667	0.6054	0.8052	0.7698
Group size (Exp1: JND)	0	18.8428	20	0.5445	0.1667	0.7533	1.0864	1.0386
Group size (Exp2: PSS)	0.0002	16.1255	5	**0.0066**	0.3333	0.6522	0.7263	0.7034
Group size (Exp2: JND)	0.0216	7.1554	5	0.2099	0.3333	0.8051	0.9307	0.9014
Group size (Exp1': PSS)	0	29.6853	20	0.0808	0.1667	0.5894	0.7633	0.7334
Group size (Exp1:' JND)	0	34.6917	20	**0.0242**	0.1667	0.5677	0.7271	0.6987

LB = lower.bound, GG = Greenhouse-Geisser
HF = Huynh-Feldt-Lecoutre, CM = Chi-Muller

Table S2. The post hoc t-test for the PSS for the group size in Experiment 1.

Pair	Diff	t(16)	p value
N=2-N=3	-0.019	5.366	0.001
N=2-N=4	-0.022	6.025	0.000
N=2-N=5	-0.024	6.268	0.000
N=2-N=7	-0.025	5.948	0.000
N=2-N=10	-0.030	6.273	0.000
N=2-N=20	-0.026	5.284	0.001
N=3-N=4	-0.003	1.223	1.000
N=3-N=5	-0.005	2.047	0.632
N=3-N=7	-0.006	2.119	0.551
N=3-N=10	-0.011	3.271	0.072
N=3-N=20	-0.007	1.796	0.914
N=4-N=5	-0.003	1.073	1.000
N=4-N=7	-0.003	1.435	1.000
N=4-N=10	-0.008	3.265	0.072
N=4-N=20	-0.005	1.323	1.000
N=5-N=7	0.000	0.108	1.000
N=5-N=10	-0.006	2.176	0.493
N=5-N=20	-0.002	0.512	1.000
N=7-N=10	-0.005	1.551	1.000
N=7-N=20	-0.002	0.480	1.000
N=10-N=20	0.004	1.255	1.000

Table S3. The post hoc t-test for the PSS for the group size in Experiment 2.

Pair	Diff	t(19)	p value
N=2-N=3	0.033	1.994	0.182
N=2-N=4	0.030	1.716	0.308
N=2-N=5	0.048	3.958	0.005
N=3-N=4	-0.004	0.267	0.792
N=3-N=5	0.014	1.103	0.743
N=4-N=5	0.018	1.209	0.743
